# Differences of use between paliperidone palmitate 3 month and paliperidone palmitate 1 month in real practice, with psychotic patients

**DOI:** 10.1192/j.eurpsy.2022.1886

**Published:** 2022-09-01

**Authors:** P. Escobedo-Aedo, J. Merayo-Cano, L. Mata Iturralde, L. Muñoz Lorenzo, S. Ovejero, S. Sánchez Alonso

**Affiliations:** Hospital Universitario Fundación Jiménez Díaz, Psychiatry, Madrid, Spain

**Keywords:** Paliperidone palmitate, severity, Long-acting inyectable, Treatment

## Abstract

**Introduction:**

Paliperidone palmitate 1-month (PP1M) is a Long-acting injectable antipsychotic formulation, approved for the treatment of schizophrenia and schizoaffective disorder. Recently, paliperidone palmitate 3-months (PP3M) formulation was introduced, which maintains stability while offering a longer dosing interval for the maintenance treatment in patients previously treated with PP1M. Despite of this, many patients are treated with PP1M without transition to PP3M.

**Objectives:**

To identify variables explaining maintenance of PP1M treatment instead of going to PP3M. We hypothesize that more severe patients are delayed in transition to PP3M because of expectation to complete stabilization.

**Methods:**

A descriptive analysis of 123 patients, diagnosed with psychotic disorders, on treatment with paliperidone palmitate 1 month or 3 months, was performed. Age, sex, type of paliperidone treatment, hospitalizations after the initiaton of treatment, years since diagnosis, polytherapy and toxic habits were some of the variables measured and compared between both groups (PP1M and PP3M).

**Results:**

Most of patients (63,41%) were on PP3M. Both groups shared characteristics like male sex predominance, schizophrenia as the most common diagnosis, having a recent onset diagnosis, same frequency of polypharmacy and same pattern of drug consumption. There was a slight difference between both groups regarding severity. PP1M and PP3M showed respectively 33% and 16,7% of admissions after initiation.

**Conclusions:**

No clear pattern determines less transition to PP3M from PP1M. No statistical difference was found except form the difference found in admission after change of treatment (to PP1M or PP3M), which could reflect influence of severity in treatment. Future research is needed in order to better elucidate this association.

**Disclosure:**

No significant relationships.

